# Integrated Insights into Metabolic and Bariatric Surgery: Improving Life Quality and Reducing Mortality in Obesity

**DOI:** 10.3390/medicina61010014

**Published:** 2024-12-26

**Authors:** Ruxandra-Cristina Marin, Andrei-Flavius Radu, Paul Andrei Negru, Ada Radu, Denisa Negru, Raluca Anca Corb Aron, Teodora Maria Bodog, Ruxandra Florina Bodog, Paula Bianca Maghiar, Roxana Brata

**Affiliations:** 1 Doctoral School of Biological and Biomedical Sciences, University of Oradea, 410087 Oradea, Romania; marin.ruxandracristina@student.uoradea.ro (R.-C.M.); adaradu@uoradea.ro (A.R.); miculas.denisaclaudia@student.uoradea.ro (D.N.); bodog.teodoramaria@student.uoradea.ro (T.M.B.); bodog.ruxandraflorina@student.uoradea.ro (R.F.B.); 2 Department of Preclinical Disciplines, Faculty of Medicine and Pharmacy, University of Oradea, 410073 Oradea, Romania; raluca.aron@didactic.uoradea.ro; 3 Department of Pharmacy, Faculty of Medicine and Pharmacy, University of Oradea, 410028 Oradea, Romania; 4 Department of Surgical Disciplines, Faculty of Medicine and Pharmacy, University of Oradea, 410073 Oradea, Romania; badea.paula.bianca@didactic.uoradea.ro; 5 Department of Medical Disciplines, Faculty of Medicine and Pharmacy, University of Oradea, 410073 Oradea, Romania; brata.roxanadaniela@didactic.uoradea.ro

**Keywords:** bariatric surgery, obesity, metabolic syndrome, Roux-en-Y gastric bypass, laparoscopic sleeve gastrectomy, biliopancreatic diversion with duodenal switch

## Abstract

Metabolic and bariatric surgery (MBS) is an effective intervention for patients with severe obesity and metabolic comorbidities, particularly when non-surgical weight loss methods prove insufficient. MBS has shown significant potential for improving quality of life and metabolic health outcomes in individuals with obesity, yet it carries inherent risks. Although these procedures offer a multifaceted approach to obesity treatment and its clinical advantages are well-documented, the limited understanding of its long-term outcomes and the role of multidisciplinary care pose challenges. With an emphasis on quality-of-life enhancements and the handling of postoperative difficulties, the present narrative review seeks to compile the most recent findings on MBS while emphasizing the value of an integrated approach to maximize patient outcomes. Effective MBS and patients’ management require a collaborative team approach, involving surgeons, dietitians, psychologists, pharmacists, and other healthcare providers to address not only physiological but also psychosocial patient needs. Comparative studies demonstrate the efficacy of various MBS methods, including Roux-en-Y gastric bypass and laparoscopic sleeve gastrectomy that may considerably decrease morbidity and mortality in individuals with obesity. Future studies should target long-term patient treatment, and decision making should be aided by knowledge of obesity, comorbidity recurrence rates, and permanence of benefits.

## 1. Introduction

Individuals who find it difficult to get rid of extra kilograms with diets and/or exercise, as well as those with a body mass index (BMI) of 35 or above and related conditions such metabolic syndrome, diabetes, hypertension, and dyslipidemia, are candidates for metabolic and bariatric surgery (MBS). Patients with a BMI above 40 but no related conditions are also considered [1]. But MBS procedures alter the digestive tract, primarily the stomach and occasionally also the small intestine. The bariatric surgical techniques control the number of calories a person can ingest and absorb. Additionally, they may lessen the signals of hunger that reach the brain from the digestive system [2]. Techniques used in MBS affect the energy balance by significantly lowering food intake and forcing the body to use stored energy. Reducing energy deposits results in decreased adipose tissue, weight loss, a return to normal appearance, a major improvement in vital functions, and the elimination or marked amelioration of obesity-related disorders. After surgery, the patients should lose weight gradually and reach a healthy weight in eight to twelve months [3].

A 1991 consensus meeting at the National Institutes of Health in the United States resulted in the international guidelines for MBS. Even back then, it was known that there were significant long-term dangers associated with early bariatric surgeries like jejunoileal bypass. On the other hand, the notion of treating obesity surgically was mostly dismissed. Later on, a consensus panel determined that vertical banded gastroplasty and Roux-en-Y gastric bypass (RYGB) are safe and effective for persons with a BMI between 35 kg/m^2^ and 40 kg/m^2^ [4].

After a period of thirty years since that moment, two prominent international organizations, the American Society for Metabolic and Bariatric Surgery (ASMBS) and the International Federation for the Surgery of Obesity and Metabolic Disorders (IFSO), agreed there is a need for some updates of the indications for MBS. They reviewed the scientific research, or, if there was a lack of information, a Delphi survey of experts was performed. The newest recommendations are included in Table 1 [5].

Obesity is unquestionably a serious public health issue [6]. A total of 650 million people worldwide, meaning 13% of the population, are thought to be obese [7]. Although there are numerous causes of obesity, it is true that the proportion of individuals with obesity is rising steadily. The World Health Organization (WHO) reports that since 1980, in several European countries, the obesity rate has increased three times, with 50% of the population being overweight or obese. Moreover, it is anticipated that a sizeable fraction of the world’s population—exactly 60%, or 3.3 billion people—may experience the consequences of excessive weight gain by 2030 [2]. The WHO’s most recent report, from May 2022, states that 60% of Europeans are overweight or obese. The consequences of the obesity pandemic are emphasized in the paper, especially when it combines with the COVID-19 pandemic to form a twin pandemic that raises morbidity and mortality. Obesity is a complex disease that has reached pandemic proportions [8].

The situation in Romania is just as alarming as it is worldwide. An observational study conducted in Oradea, Romania, which included 900 people between the ages of 18 and 65, evaluated the impact of adiposity risk variables on the prevalence of overweight and obesity. It also found the prevalence of these risk factors. In contrast to 2010 when it was estimated to be around 10% [9], the outcomes showed that 29.56% of the population was overweight and 21.33% was obese [10].

In 2018, a significant rise in the rate of childhood overweight to 18.5% was observed. Furthermore, parents and grandparents still enthusiastically use the term “chubby and beautiful” in our nation. Many of them do not understand that being overweight or obese is a problem and, as a result, are unwilling to actively engage in weight loss exercises [11].

At any age, compared with men, women have a higher probability to be obese. Between the ages of 50 and 65, it reached its pinnacle, following which it started to progressively fall. The age prevalence of obesity increased from approximately 5% to 14.0%, in the interval between 1980 and 2019. The two countries with the highest rates of obesity among their populations are the USA and Russia. The regions with the greatest prevalence of obesity are North America and Europe [12].

Obesity is one key part of metabolic syndrome (MS), which commonly coexists with other MS components like type 2 diabetes mellitus and arterial hypertension [13]. Overweight also raises the chances of cardiovascular illnesses, certain cancers, and early mortality. MBS is a useful instrument in the management, therapy, and prevention of several metabolic diseases associated with obesity, including diabetes and fatty liver disease. Surgery to lose weight, though, is not an easy and rapid method. Continuous lifestyle changes must be performed after meticulous planning for them to be effective. It has also been shown that being overweight is linked to depression and a much worse quality of life [14].

Research has indicated that individuals who are obese commonly experience eating disorders, anxiety, sadness, and social stigma. They also frequently exhibit a skewed body image [15,16]. Studies have also shown how people with obesity, and particularly those who are extremely obese, have deficits in various other categories as well as quality of life. It is thought that these limitations have a major influence on the decision to have MBS. Before achieving their ideal weight loss, MBS individuals frequently describe notable gains in these and other psychosocial well-being [17]. Because it has been demonstrated to generate better therapeutic control of the diseases linked with obesity than medication alone and to enhance the quality of life for obese people, MBS is an essential treatment alternative in the problems outlined above.

MBS requires a comprehensive, multidisciplinary approach in addition to surgical procedures for addressing the dietary and psychosocial needs of the patient. One of the main components of this multidisciplinary approach is the collaborative work of a team of different healthcare professionals. Surgeons, nurses, doctors from other specialties, clinical psychologists, clinical pharmacists, nutritionists, and other pertinent professionals usually make up this team. The goal of their combined experience is to improve the patient’s overall well-being and the surgical outcome.

The present narrative review aims to consolidate and update the current knowledge on the management of therapy and patient recovery following MBS, with a dual focus on the substantial quality-of-life improvements observed in patients and the potential risks, long-term changes, and complications associated with this intervention. By integrating recent findings, this work contributes to a more nuanced understanding of MBS outcomes, offering valuable insights for advancing both patient care and the scientific literature in metabolic and surgical management of obesity.

## 2. Research Methodology

**Figure 1:**
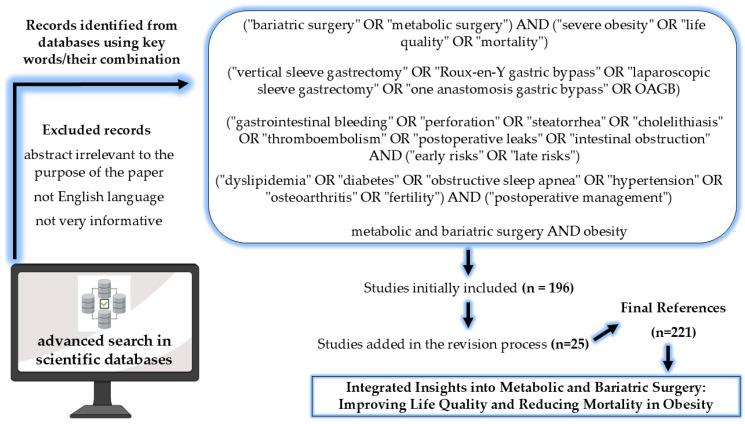
Algorithm of the selection methodology for the bibliographical sources evaluated and cited in this paper.

The present research methodology was based on the selection of bibliographic sources from scientifically relevant databases (i.e., PubMed, Embase, Google Academic, Web of Science, ScienceDirect, etc.) based on a clearly defined advanced search algorithm utilizing Boolean operators and their evaluation for updating in a comprehensive manner with the latest and most relevant scientific data in the field of MBS (Figure 1). Furthermore, MeSH and Emtree controlled vocabularies were also used in the databases that offered this possibility.

Journal articles, books, and web pages of regulatory and evaluation bodies in the field were used as bibliographic sources to update data on the pathophysiological mechanisms of obesity; types and characterization of MBS, with emphasis on the RYGB, laparoscopic sleeve gastrectomy (LSG), and one anastomosis gastric bypass (OAGB); factors influencing the surgical decision, such as BMI and age extremes; the impact of MBS on obesity comorbidities and their postoperative management; the risks associated with MBS, such as early and late complications evaluation and weight regain after MBS; and the psychological implications and body image after MBS.

A total of 196 bibliographical references were selected, evaluated, and initially cited in this paper as validation of the presented data. Furthermore, in the revision process 25 more references were included, totalling 221 references.

## 3. Pathophysiological Foundation of Obesity

Obesity, often known as excessive or extra body fat, is a complicated chronic illness that affects health, shortens life expectancy, and increases the chance of developing chronic illnesses [18]. The World Obesity Atlas 2023 study assessed that almost 40% of the global population have a BMI over 25 kg/m^2^, making them overweight or obese. The World Obesity Federation’s World Obesity Atlas 2023 provides projections for the prevalence of obesity and details on the rising financial expenses linked to overweight and obesity for the years 2020–2035. It is concerning to note that by the conclusion of this era, over 50% of the world’s population is predicted to have a high BMI, and 1 in 4 individuals will be obese, up from 1 in 7 currently [19].

Controlling energy intake and consumption allows for the achievement of energy balance. From this point of view all calories are equal and a calorie is really a calorie. Considering the pathophysiology of obesity-related comorbidities and, besides this purely energetic consideration, not all calories are equal [20]. While the mechanisms governing energy balance are largely agreed upon, the ideal nutritional composition is a matter of debate and misunderstanding [21].

Most scientists consider that obesity or body weight is deliberately controlled or maintained. There are two distinct but related mechanisms at play in the physiology of obesity: (1) a continuous state of positive energy balance (intake vs. expenditure) and (2) a reset of the body weight “set point” to a higher value. This latter process explains the tendency of weight loss to be gained again over time, if it was obtained due to dietary and/or lifestyle modifications. This is a significant barrier to the effective treatment of obesity [22]. New information reinforces the idea that obesity is an illness and absolves the individual of responsibility in favor of physiology [23].

One of the main functions of the biomedical hypothalamus is to recognize and translate constraints in the short- and long-term nutrient supply into behavior. Therefore, two distinct categories of biologically different neurons, called POMC/CART and AGRP/NPY, are sensitive to both brain signals from the brainstem and vagus nerve that reflect the nutritional status of the gut, as well as hormones and moving metabolic products that signal the availability of energy, such as leptin, ghrelin, insulin, and glucose [22].

While the precise function of ghrelin in the development of obesity remains unclear, a comprehensive understanding of how elevated ghrelin levels cause hunger might transform obesity management, if not outright “treatment”. Numerous studies have revealed that obese groups exhibit lower postprandial suppression of ghrelin as opposed to controls with normal BMI; this is a new and plausible reason for the obese individual’s higher food consumption, as they continue to feel hungry even after eating [24]. According to this circumstance, the issue is not ghrelin sensitivity or oversensitivity, but rather the overproduction of ghrelin independent of meal consumption [25].

Clinical phenotypes linked to deficits in LEPR and leptin are strikingly similar. Patients have a normal weight at birth, but throughout the first few months of life, they rapidly gain a significant amount of weight, reaching the extremes of obesity. Moreover, body composition analyses showed that these conditions are distinguished by the predominant accumulation of fatty tissue, with the limbs and trunk displaying disproportionately high amounts of subcutaneous fat [26]. Numerous genetic chromosomal regions have been associated with obesity, and findings from genome-wide association studies suggest that being overweight is inherited [22].

Overweight and obesity are categorized by the WHO, with overweight being defined as BMI > 25 kg/m^2^ and obesity as BMI > 30 kg/m^2^. Since BMI is easy to calculate, it is the most frequently used initial step in evaluating the degree of overweight or obesity, even if it is not the most reliable measure of excess fat [27]. Obesity raises the likelihood for acquiring certain diseases, such as depression, sleep apnea, non-alcoholic fatty liver disease, osteoarthritis, diabetes mellitus, hypertension, coronary heart disease, and heart failure [28].

All age groups and both sexes are impacted by obesity in wealthy nations, but people with lower socioeconomic status (SES) are more susceptible. Obesity primarily affects mature rich individuals in developing countries, particularly metropolitan women [29].

From an environmental perspective, a population’s ability to develop obesity depends on its economic success and financial resources, yet obesity can also occur in impoverished populations. Supermarkets, fast food restaurants, parks, transportation, and sociocultural and economical elements are among the other important external factors that lead to obesity [30].

One of the most effective and tried-and-true methods of treating obesity and its linked conditions is MBS. Numerous studies discovered in the literature demonstrate that the surgical anatomic manipulation associated with gastric bypass causes significant physiological and metabolic alterations in several organs. Nonetheless, little is still known about the complex physiological relationships that control energy homeostasis, appetite stimulation, and the causes of obesity in humans [31].

## 4. Description and Types of MBS

In cases of extreme obesity, MBS is becoming a viable choice after all nonsurgical weight loss measures have been tried and tested. Apart from its immediate effect on weight loss, MBS enhances numerous health parameters in the aftermath of surgery. When nonsurgical measures are ineffective in controlling the patient’s weight, then surgical intervention becomes essential [32]. But not every patient loses enough weight or has their comorbidities resolved, and there is always a chance of perioperative problems. To estimate the chance of success and select the best surgical method for this goal, accurate preoperative evaluation is crucial [33].

BS treats severe obesity using a range of surgical procedures. A recent study selected data items from different studies, from 24 countries, in order to define the types of bariatric surgical interventions being performed, the demographics of obese patients undergoing MBS, and perioperative safety factors. According to the statistics, sleeve gastrectomy (SG) was the most common primary surgical technique (62.5%), followed by RYGB in second place (28.5%), and OAGB in third place (4%), out of all the weight reduction surgery operations [34].

More than 90% of procedures were carried out using a laparoscope [35]. According to a 2007 clinical study, bariatric procedures were divided into malabsorptive, restrictive, and a combination of the two surgical procedures [36].

The restriction is a part of every procedure. Chime reaches the small intestine more quickly than it would in normal anatomy because the stomach’s capacity is reduced to varying degrees and the gastric emptying time is accelerated. By rewiring the stretch reflexes that link the gut and the brain, anatomical alterations in each BS encourage the patient to consume fewer meals. This phenomenon is more severe just after the procedure, lasts for months, and then fades with time. Malabsorption is the second mechanism of action. With sleeve gastrectomy it does not occur. However, it is evident in the cases of OAGB and RYGB. A section of the small intestine, which absorbs the most nutrition, is bypassed throughout each of these procedures. Restrictive procedures include laparoscopic adjustable gastric banding, vertically banded gastroplasty, and LSG. Combined procedures, both restricted and malabsorptive operations such as RYGB and OAGB are the common ones [37]. Nowadays, researchers recognize that this classification offers an incomplete explanation for weight loss.

SG is considered to be a restricted procedure. A smaller, sleeve-shaped stomach is left behind after about 80% of the stomach is surgically excised during a sleeve gastrectomy. Both restriction (making the stomach smaller) and intricate metabolic modifications, such as adjustments to gastrointestinal hormones, are how this operation functions. As a result, there is less hunger and an earlier sense of fullness or satiety after eating [38].

RYGB was considered a mixed technique, both restricted and malabsorptive. This procedure acts by forming a little gastric pouch. In that way, food is diverted from the majority of the stomach and a tiny section of the small intestine. If necessary, this operation can be reversed. Its exact mechanism of action entails intricate hormonal relationships that control blood sugar level and satiety, or the sensation of fullness [39].

OAGB is also a combined procedure [40]. It delivers an excellent quality of life with a controlled risk of complications and has shown extraordinary efficiency in alleviating comorbidities connected to obesity [41].

**Figure 2:**
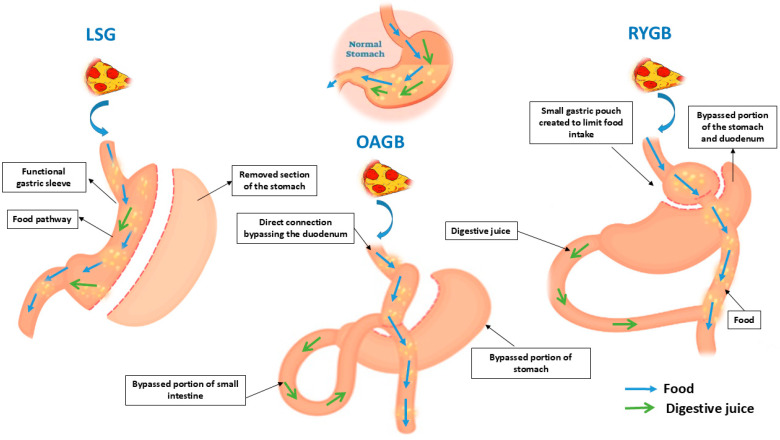
Schematic description of the three most commonly used bariatric procedures. LSG, laparascopic sleeve gastrectomy; RYGB, Roux-en-Y gastric bypass; OAGB, one anastomosis gastric bypass.

Figure 2 illustrates a schematic representation of the three most frequently performed bariatric procedures.

### 4.1. The Restrictive–Malabsorptive Roux-en-Y Gastric Bypass (RYGB)

In 1994, the first laparoscopic version of the RYGB was carried out. The technique begins similarly to a sleeve gastrectomy, with the abdominal cavity being entered with the use of the Hassan or Optiview techniques to place a trocar above the umbilicus. Following the appropriate inflation, four to five more trocars are directly visualized with a 30-degree laparoscope. Again, to preserve the liver and related tissues and to enhance gastric exposure, a retractor is frequently used. The formation of the stomach pouch comes next, following appropriate dissection and mobilization. The pouch is constructed as a closed, 20–30 cc pouch by firing several GIA linear staples to the proximal stomach. Food is therefore unable to pass through the esophagus and into the residual stomach. The duodenum and proximal jejunum remain attached to the remaining stomach, forming the afferent or biliopancreatic (BP) limb [42].

Significant body weight loss is the outcome of RYGB due to the decreased eating, higher energy expenditure, nutritional restrictions, and maybe changed metabolic efficiency [43]. After a year, weight loss equals roughly 77% of additional body weight, with a significant degree of remission for preexisting multiple medical conditions, particularly diabetes mellitus [44].

### 4.2. Laparoscopic Sleeve Gastrectomy (LSG)

LSG is a non-reversible treatment that uses partial gastrectomy to permanently reduce the size of the stomach while keeping the stomach’s minor curvature and pylorus intact. The bowel is longitudinally resected in LSG starting against the Latarjet nerve and finishing at the angle of His, following the bigger curve from the antrum. It is a minimally invasive weight reduction method that forms the stomach into a tubular structure by shrinking its size using an endoscope. Losing weight and reducing caloric consumption are possible outcomes of this process. The segmentation of the gastrocolic and gastrosplenic ligaments near the stomach allows for the split of the blood supply of the stomach’s larger curvature, which is the first step in the process. To fully resect the gastric fundus, which houses the stomach’s ghrelin-secreting cells, the greater curvature must be released all the way to the left crus of the diaphragm. The longitudinal gastrectomy, which reduces the stomach to a narrow tube by “sleeving” it, is the second phase in the process [45].

To provide accurate calibration and prevent gastric stenosis after a gastric plastic, a nose–gastric tube is utilized. There is disagreement over the best place to begin a gastric bypass and the appropriate nose–gastric tube caliber. Starting the gastrectomy 10 cm in front of the pylorus was Ganger’s recommendation [46].

Through the activation of a satiety mechanism, the procedure causes and maintains weight loss. Changing the amount of fluid in the band allows for an appropriate filling. Food is not physically restricted above the precisely adjusted band; rather, food boluses transit through the band temporarily and the constant pressure of the optimally filled band on the stomach wall results in early satiety and decreased appetite [47].

Without actually limiting the amount of food consumed, the band’s effects on esophageal and proximal stomach function seem to trigger a satiety signal that is sent to CNS satiety centers via the vagus nerve [48].

### 4.3. One Anastomosis Gastric Bypass (OAGB)

Dr. Robert Rutledge performed the OAGB for the first time in the United States in 1997, but from that moment the procedure has undergone important changes. In order to create a gastric pouch, the stomach is first divided horizontally beneath the farthest branches of the anterior and posterior vagal trunks. This is followed by a vertical split that avoids the angle of His. More specifically, the stomach is cut off at the lesser curvature, three to five cm from the pylorus. Using a bougie size of 36–38 Fr, an incision is made proximally to the left side of the cardia to form a tube-shaped stomach with a volume of about 100 mL. The distance between the gastrojejunostomy and the Treitz ligament is 200 cm. The biliopancreatic limb is hanging by 3–5 cm, and the anastomosis has a diameter of 3–4 cm. The goal is to guarantee that food can pass freely from the upper input to the output. The physiology of postgastrectomy syndrome is caused by this structural alteration, which quickly empties the stomach contents into the midjejunum. As a result, patients may naturally steer clear of high-calorie and high-fat items in favor of smaller, lower-fat, and lower-sugar meals. The primary principles of the OAGB method are the construction of a loop gastrojejunostomy that avoids the proximal small bowel (malabsorptive component) and the creation of a modestly large gastric pouch (restrictive component) [49].

### 4.4. Comparing the Three Procedures—Maintaining the Weight Lost and the Impact on Associated Diseases

In a retrospective cohort study, 261 Korean individuals with obesity who had undergone various bariatric operations had their medical records reviewed. The study demonstrated comparable outcomes when comparing the three treatments, taking into account the weight lost and the reduction in associated diseases, including dyslipidemia, hypertension, and diabetes. RYGB, however, showed a greater rate of resolution of comorbidity. At 18 months, the weight reduction outcomes from the three operations were comparable (weight loss was a bit over 50% for SG, 61.0% for LAGB, and approximate 70% for RYGB). Patients with RYGB had higher rates of diabetes, hypertension, and dyslipidemia remission (65.9%, 63.6%, and 100% of patients, respectively) [50].

In 2020 a meta-analysis was performed based on 20 studies, which included 2917 patients. The outcomes of RYGB and LSG did not significantly differ taking into account the excess weight loss. Furthermore, there were no appreciable variations in the mid- and long-term weight loss outcomes between the similar groups. Similarly, there was not much of a difference in the way type 2 diabetes was resolved. RYGB was better than LSG at treating dyslipidemia, hypertension, and gastroesophageal reflux disease [51].

OAGB provides comparable, if not better, outcomes when talking about weight loss and metabolic enhancements than RYGB. According to the findings of a comparative study conducted in 2014, patients receiving OAGB had a marginally greater estimated body weight loss at 24 months (72% vs. 68%) than patients undergoing RYGB. Additionally, the outcomes also showed that the OAGB group had an 84% T2D remission rate, whereas the RYGB group had a 78% rate [52].

Compared to RYGB, OAGB also has a shorter operating duration and a comparatively lower rate of complications. OAGB is said to have a lower rate of postoperative complications, including anastomotic leaks (0.5% vs. 1.2% in RYGB), and a shorter operating time (mean of 85 min vs. 120 min for RYGB). Furthermore, the OAGB procedure’s simplicity—it only requires one anastomosis—contributes to its safety profile and decreased risk of complications including internal hernias [53].

Another clinical study sought to compare the outcomes of two bariatric operations after three years. The study included 55 patients, and after the three-year follow-up, the outcomes were similar for the two procedures in terms of patients’ BMI and their weight loss. Also, the remission of comorbidities and the percentage excess weight loss had similar results. The conclusions of the researchers were that both RYGB and OAGB are effective when speaking about morbid obesity [54].

When comparing OAGB with LSG, a meta-analysis from 2024 included all studies which had been printed between 2015 and 2022 and enrolled over 6000 patients. It showed a statistically significant result (*p* < 0.05) in favor of OAGB in terms of remission for dyslipidemia, hypertension, diabetes mellitus, and bleeding. On the other hand, OAGB made GERD and leaking more common (*p* < 0.05) [55].

A review published in 2017 analyzed the results from 17 studies which included almost 7000 patients who had undergone an OAGB intervention or an LGS one. According to that review, the OAGB group experienced reduced mortality, a shorter mean hospital stay, remission of comorbidities, and higher weight loss. The two methods had comparable rates of leaks and intra-abdominal bleedings [55].

## 5. Factors Influencing Surgical Decision Making

BMI, medical history, obesity-related medical conditions, surgical risks and patient preferences, surgeon experience and skill, and patient dedication to dietary and lifestyle modifications are some of the major factors that affect surgical decision making [56].

### 5.1. BMI

The leadership of ASMBS, IFSO, and other international professional organizations have gathered to produce statements of scientific knowledge regarding metabolic and bariatric invasive procedures and its guidelines, in light of the notable developments in our knowledge of obesity as a disease, its treatment generally, and MBS in particular [57].

BMI is the primary indicator. When MBS is necessary, it is determined by the existence of associated diseases and body mass index (BMI). Those with a BMI of 40 kg/m^2^ or more, no concomitant associated diseases, and for whom MBS would not provide an unreasonable danger should be considered candidates for one of the aforementioned surgeries. Those who fit any of the following descriptions would also be eligible for surgery: a BMI of 35 kg/m^2^ or above; one or more severe associated condition (type 2 diabetes, hypertension, hyperlipidemia, GERD, asthma, venous stasis disease, obstructive sleep apnea (OSA), non-alcoholic fatty liver disease (NAFLD), severe urine incontinence, debilitating arthritis, or a markedly diminished quality of life). Patients who have metabolic syndrome or diabetes with a BMI between 30 and 34.9 kg/m^2^ may also think about having weight loss surgery, though there is not enough data to demonstrate lasting benefits in these patients [58].

### 5.2. Age Extremes

Table 2 presents a summary of expectations and risks associated with MBS in extreme age groups, specifically older adults (>70 years) and pediatric/adolescent patients (<18 years). For older patients undergoing LSG, significant weight loss and co-morbidity remission were noted, though with an increased risk of complications post-surgery. In younger patients undergoing Roux-en-Y gastric bypass (RYGB), substantial and sustained improvements in cardiovascular and metabolic health were achieved but with associated risks, including nutritional deficiencies, hernias, and gastroesophageal reflux. These findings highlight distinct considerations for surgical outcomes based on age.

## 6. Physiological Changes to the Digestive System Following MBS

Following MBS, several aspects of gut physiology alter, including sensation of taste, meal pattern and duration, intestinal transit time (ITT) and gastric emptying (GE), gut hormone release, bile acid (BA) metabolism, and microbiota. Because LSG modifies dietary transit time and eliminates part or all of the cells that produce the potent orexigenic hormone ghrelin, it may be more effective than other restrictive techniques. This could be the reason behind the physical appearance of LSG as an exclusively restrictive treatment. But its mode of action is probably going to be far more complicated (Table 3) [65].

### 6.1. Factors Potentially Influencing Intake, Digestion, and Absorption

**Figure 3:**
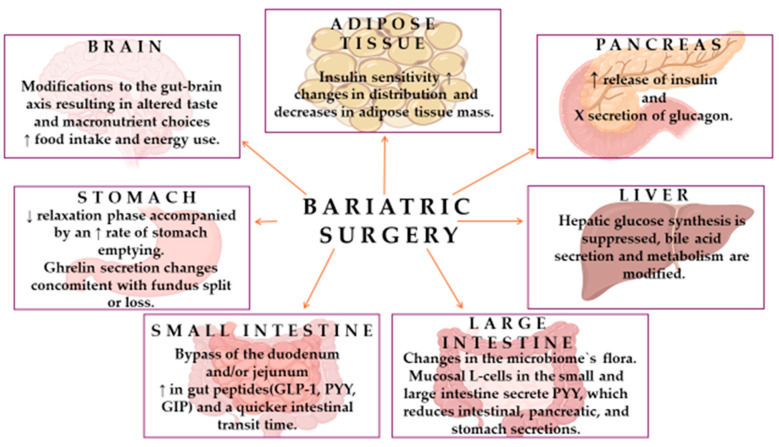
Organ systems targeted by MBS and the associated physiologic changes.

Several organ systems are targeted by MBS: brain, stomach, small and large intestines, liver, pancreas, adipose tissue, and muscle tissue (Figure 3).

Fast weight loss occurs in the first few months after MBS. This phenomenon is due to an important, inadvertent loss of muscle mass and fat-free mass [104]. Protein consumption is often and significantly decreased following MBS, particularly during the initial months following the procedure. This is mainly due to stomach resistance to meals high in protein. On the other hand, it is believed that consuming enough protein can prevent the loss of muscle mass during slimming down [105].

Based on current guidelines, protein consumption after MBS should be set between 60 g/day and 1.5 g/kg; in certain cases, higher protein intake (up to 2.1 g/kg ideal body weight per day) may be necessary [106,107]. The prevention, recognition, and therapy of a lack of vitamins and minerals are the primary topics of ongoing monitoring following MBS. Following MBS, all patients should be administered a routine daily multivitamin and mineral prescription based on the type of treatment and current guidelines [107].

While MBS has produced many health benefits, such as the remission of diabetes, it is also linked to a consequence that is currently underappreciated: postprandial reactive hypoglycemia. Because there is disagreement over the definition and diagnosis of MBS-related hypoglycemia, it is unclear how common the disorder actually is [108].

It is crucial to measure the degree of macronutrient malabsorption even if it is not a primary cause of weight loss following MBS for a number of reasons. First, understanding the degree of malabsorption of macronutrients may help to prevent undernutrition by improving dietary guidance for patients following surgery [109]. Due to postoperative vomiting, reduced food intake, food aversion, decreased stomach secretions, and bypass of absorption surface areas MBS patients are more likely to experience nutritional deficiencies [110].

### 6.2. Prevalent Gastrointestinal Symptoms: Preventive Strategies and Interventions

Compared to other nonsurgical therapies, MBS has shown a better success rate in treating selected individuals with obesity. As with any medical procedure, there is a chance of postoperative complications or complaints from patients, which can range from 2 to 10% as studies demonstrated [111,112]. After gastric surgery, individuals may develop the phenomenon known as “dumping syndrome”. The fast passage of hyperosmolar chyme from the stomach into the small intestine causes the patient’s gastrointestinal physiology to change, which has undesirable consequences [113].

Usually happening an hour or less after eating, the dumping syndrome is unrelated to hypoglycemia. It is believed that the contraction of the plasma volume caused by the fluid moving into the digestive system causes it. Tachycardia, stomach pain, diaphoresis, nausea, vomiting, diarrhea, and occasionally hypoglycemia can all be symptoms of dumping syndrome. The late dumping syndrome is brought on by hypoglycemia, which is driven on by hyperglycemia and the spike in insulin that follows, two to three hours after a meal. One important issue that arises in individuals with RYGB and in those who take high doses of simple carbohydrates is dumping syndrome. To reduce the likelihood of this condition occurring, patients should avoid eating a lot of simple sugar in their meals. They can replace them with a high-protein, high-fiber diet. It is recommended to eat salads and vegetables but not to consume alcohol or other beverages [114]. A total of 20% of patients undergo symptoms of dumping syndrome after consecutive vagotomy and pyloroplasty; however, this proportion rises to 40% and peaks at 50% after a sleeve gastrectomy and RYGB. Dump syndrome is often encountered in the first few months after RYGB [115].

The most prevalent adverse reactions following MBS are nausea and vomiting, which are typically caused by unhealthy eating practices and a disregard for the gastroplasty diet recommendations, which call for eating undisturbed, chewing food thoroughly, never drinking during meals, and waiting two hours before drinking after consuming solid food. A different diagnosis must be considered if these symptoms are linked to epigastric pain, severe dehydration, or cannot be explained by dietary errors. Anastomotic ulcers, both with and without stomal stenosis, are some of the most common adverse effects that induce vomiting and nausea among individuals after a gastric bypass procedure. There is a documented incidence of 3% to 20% for ulceration or stenosis at the gastrojejunostomy of the gastric bypass [116].

Food intolerance, which is primarily experienced in the initial months after MBS, and is brought on by nausea, vomiting, and regurgitation, has been documented to affect 35–65% of people [117]. Deficits in protein and micronutrients have been demonstrated to be triggered by the ensuing changes in eating habits and meal patterns [81]. In this particular situation, the most severe micronutrient shortfall has been reported to be thiamine insufficiency coupled with protracted vomiting and nausea [118].

After surgery, there was a considerable shift in fecal consistency. Following BPD and RYGB, loose stools and diarrhea were more common (*p* < 0.001). Furthermore, there was a higher frequency of foul-smelling flatus that interfered with social life following BPD compared to RYGB or AGB (*p* < 0.05). Additionally, the frequency of flatus rose following BPD and RYGB [119].

After MBS, gastroesophageal reflux disease, or GERD, is another major complaint. GERD prevalence has been connected to LSG. After RYGB, patients who experience recurrent GERD symptoms should have a computed tomography (CT) scan or upper gastrointestinal (GI) contrast study performed. If weight gain is seen, the patient should be sent to a bariatric surgeon. This might point to a gastro-gastric rupture connecting the remaining stomach to the gastric pouch. The most effective test to rule out other esophagus gastroduodenal diseases is upper endoscopy. Esophageal problems such as esophagitis, peptic stricture, Barrett’s metaplasia, esophageal cancer, and other pulmonary difficulties can be linked to GERD. The three groups’ BAROS scores were relatively comparable. However, in BPD and RYGB, the quality of life as determined by BAROS was inversely correlated with the severity rating of flatulence [120].

Following LSG, dysphagia and postoperative emesis are frequent complications, but not much research has looked at the causes and management of both conditions at the same time. Due to the sharp increase in LSG procedures, individuals with a history of LSG will often consult gastroenterologists for dysphagia. Treatment options for gastric sleeve constriction include endoscopic hydrostatic balloon dilatation, pneumatic achalasia balloon dilation, surgery, and conservative medicinal therapy [121].

Depression and psychosomatic disorders—numerous studies have shown a connection between eating disorders and schizophrenia, depression, or anxiety disorders [122].

After losing weight, many patients typically report improved situational sadness and increased self-esteem. But treatment for depression is frequently ongoing, especially since many individuals with obesity used to eat as a coping mechanism [123].

A somatization process accompanied by symptoms of sadness and psychosomatic diseases can arise from misguided emotions. After MBS, it is critical for clinicians to acknowledge the mental component of weight loss and convince patients that their feelings are due to their smaller stomach pouch. Therapy that focuses on emotions and behavior is said to be highly beneficial [124].

Weight loss—following MBS, main clinicians should oversee all medical care while closely collaborating with other healthcare providers to fulfil the patients’ constantly changing needs. Following surgery, the patient’s general and mental health are the main focus of clinical examinations, as well as their physical activity, food intolerances, vitamin and mineral supplements, and nutritional and water consumption. Patients usually feel a lack of appetite, early satiety, and a decrease in food cravings. Every clinical visit should include a record of blood pressure, weight, weight change, biochemical testing as well as a comparison of actual and expected weight reduction [125].

## 7. The Impact of MBS on Comorbidities of Obesity

An essential component of the multidisciplinary approach to MBS is postoperative follow-up. Physicians monitor patients’ progress and evaluate the remission of comorbidities by performing routine examinations during follow-up appointments. It is teamwork. Clinicians, clinical psychologists, and nutritionists make up this team, and they are all crucial to the patient’s postoperative care [126]. Individualized testing should be performed to track the management of comorbidities such as sleep apnea, nonalcoholic steatohepatitis, hypertension, dyslipidemia, diabetes mellitus, and other conditions. Medical interventions should also be reviewed and adjusted as necessary.

The reduction in the prevalence of lipid metabolic diseases (66%), hypertension (68%), and diabetes (61%) three years following surgery is comparable to the findings of recent meta-analyses [127]. The research has demonstrated that the clinical results of MBS are greatly impacted by adherence to postoperative follow-up. A study conducted in Spain, in 2016 showed that patients who missed one or more of their follow-up medical visit during the 12-month weight loss period were not as successful as bariatric patients who strictly followed the medical controls, at 3, 6, and 12 months, *p* < 0.001 [126].

### 7.1. Postoperative Benefits of MBS over Diabetes

In the early postoperative phase, diabetic patients should have regular blood glucose testing and sliding scale insulin management. Following MBS, many diabetes patients no longer require insulin or oral hypoglycemic medications. For many, but not all, persons with severe obesity-related diabetes, RYGB is linked to a long-lasting recovery of type 2 diabetes (T2D) [128].

In the early 1980s, surgeons observed clearly that many type 2 diabetes patients who underwent RYGB experienced a return to normal of their diabetes. Pories et al. conducted retrospective single-cohort research of 298 people with RYGB who also had T2D or poor glucose tolerance. Among those patients, 91% continued to have normal fasting glucose and A1C levels, with only 4% of them discontinuing throughout a 14-year follow-up period. For longer periods of time (approximate 5 years, compared with one and a half), those who did not recovered were older (*p* < 0.001) and had a history of T2D [129].

In previous research, 767 individuals with obesity who had surgery were analyzed in comparison with a matched control group with a similar number of subjects who received standard care. At two years (0.2% vs. 6.3%) and ten years (7% vs. 24.9%) following surgery, the surgical group’s incidence was determined to be considerably lower than that of the group acting as the control [130].

More recently, a number of studies with over 800 participants in randomized clinical trials (RCTs) comparing medication and surgery for the management of T2D have been published. These studies covered patients with mild to severe T2D with a BMI between 25 and 53. All of these showed that surgical techniques were more effective than pharmaceutical treatments for achieving control of glucose levels and remission of type 2 diabetes, with a variable frequency of reduction happening at different time intervals following treatment, from one to five years. Generally, medication and surgery reduced HbA1c by 0.4% to 1.5% and 1.8% to 3.5%, respectively, for glycemic control [131,132,133,134,135].

A meta-analysis which compared the outcomes for diabetic patients undergoing MBS with those taking medication treatment showed that MBS was linked to an increased glycemic control ratio (*p* < 0.001), a smaller HbA1c rate (*p*< 0.001), and a statistically significant higher ratio of T2D remission. In the study were included 706 diabetic patients who underwent control medical visits at 12 and 36 months after the surgery [136].

### 7.2. Postoperative Hypertension Management

Although obesity and hypertension (HTN) are not necessarily linked, and dietary changes do not guarantee that blood pressure will return to normal, losing weight lowers hypertension associated with obesity. Specialists recommend that, after MBS, patients undergo weekly monitoring until their blood pressure stabilizes. It may be necessary for them to begin antihypertensive medication, albeit with modified dosages [137]. Also, it was demonstrated that 68% of patients considering MBS who are severely obese also have hypertension [138].

According to systematic evaluations, the 1-year rate of hypertension remission following MBS ranges from 43% to 83%. Patients receiving RYGB appear to have greater rates of hypertension remission (5-year RR, 1.26 [95%CI, 1.07–1.48]) when compared to patients undergoing sleeve gastrectomy; however, a five-year variations in systolic and diastolic blood pressure might be comparable among all techniques [139].

A Swedish prospective study from 2000 used a computerized matching program to match 346 gastric surgery candidates with 346 obese control people based on 18 variables. The findings showed that, initially, the surgical group’s systolic blood pressure decreased by 11.4% mm Hg and diastolic blood pressure dropped by 7.0% mm Hg over the first half a year, a time of considerable reduction in weight. Over the following half a year, there was a slower rate of weight reduction, a rise in systolic blood pressure levels, and an absence of the diastolic blood pressure decline. Long-term, systolic blood pressure did not vary during an 8-year period, even though the surgery group’s body weight significantly continued to decrease compared to that of the control group [140].

In a randomized, single-center, blinded trial, patients with HTN (using ≥2 medicines at maximal doses or >2 at medium doses) with a BMI between 30.0 and 39.9 kg/m^2^ were included. The decrease in the number of antihypertensive medicines by ≥30% with the preservation of systolic and diastolic blood pressures of less than 140/90 mmHg was more frequently met by the bariatric patient’s group (83.7%) than by the control group (12.8%), in the twelve months after the surgery. In addition, one year after surgery, almost 50% of the individuals in the MBS group and 0% in the standard therapy group had HTN remission (systolic and diastolic blood pressure < 140/90 mmHg), with prior medication withdrawal [141].

Between 2005 and 2011, a nonrandomized prospective cohort study, on 197 patients, was carried out in Spain on extremely obese subjects who experienced LSG or RYGB, with a 36-month follow-up. A total of 47.7% of hypertensive individuals continued to have HTN, but 68.1% had HTN remission one year following surgery; at three years, 21.9% of these patients had relapsed. The number of antihypertensive drugs taken before surgery was associated with a reduced remission rate in the first 12 months and a higher recurrence rate after three years. On the other hand, a higher risk of HTN recurrence after three years was correlated with a lower ratio of weight loss during the first year. Therefore, following MBS, the outcomes of a shorter-term follow-up were much higher than those of a mid- and long-term follow-up [142]. Thus, although medically induced and maintained weight loss seems to have no positive impact on arterial pressure, it does have a good effect on pulse pressure, which is a dependent predictor of cardiovascular mortality and coronary artery disease.

### 7.3. Postoperative Dyslipidemia Management

Visceral obesity and atherogenic lipid disorders are closely associated. The features that define it are low-density lipoprotein (LDL) particles, high-density lipoprotein (HDL) cholesterol, elevated triglycerides (TG), and apolipoprotein B. A total of 64% of extremely obese people who are thinking about MBS have dyslipidemia, which can show up as low HDL, elevated TG, or a combination of these [138].

Atypical lipid–lipoprotein profiles are commonly observed in individuals with extreme obesity. After MBS, there are notable improvements in the lipid–lipoprotein profile early in the postoperative phase, before weight loss, and these improvements last over time. After MBS, there may be positive effects on sensitivity to insulin, adipose tissue distribution and function, liver fat composition and activity, and lipid–lipoprotein metabolic processes, all of which contribute to the remission of dyslipidemia [143].

In a long-term cohort study involving 2348 patients, the prevalence of dyslipidemia was still lower seven years after RYGB compared to baseline (HDL, TG, and LDL, *p* < 0.001 for all) [138].

In 2021 a meta-analysis included 13 medical procedures and 2198 participants from 35 randomized controlled trials. With mean differences of −0.97 for TG, −1.98 for total cholesterol, 0.53 for HDL, and −0.94 for LDL when compared with control groups, the results showed that duodenal switch (DS) is a potential effective medical intervention for patients with severe dyslipidemia in addition to RYGB and LSG [144].

According to another meta-analysis which included over 7000 participants from observational studies and RCTs, the rate of improvement or remission of dyslipidemia was bigger with RYGB than with SG (*p* < 0.05). This was before the participants’ 3-year monitoring. After a 3-year examination, however, no statistically significant differences were found [145].

### 7.4. The Management of Obstructive Sleep Apnea Post MBS

Obstructive sleep apnea (OSA) is quite a frequent type of sleep-disordered breathing. It is defined by repeated upper airway obstruction throughout rest, which results in futile breathing attempts. One of the primary causes of OSA patients is obesity. It is advised for all individuals with this condition to lose weight since it aids in the management of therapy of obstructive sleep apnea [146]. The high rate of OSA in overweight people is correlated with the rising BMI. The incidence in those who are extremely obese varies from 55% to 100% [147].

Many patients still have mildly to moderately elevated scores following MBS, despite the fact that meta-analyses demonstrate that the procedure considerably lowers BMI and Apnea–Hypopnea Index values [148].

A review published in 2018 included 27 studies (with a total of 1169 individuals) and contained the RYGB or LSG operation. The Apnea–Hypopnea Index had a pooled mean pre-surgery score of approximate 39 events per hour and a mean score of 12.5 events per hour, after the surgery. Studies have shown that individuals with more severe obstructive sleep apnea experienced bigger score decreases. Ten studies provided pre- and post-surgery Epworth Sleepiness Scale scores; the mean SD reduction was significant, going from 11 to 5.6. A score higher than ten shows a lot of tiredness. According to the Apnea–Hypopnea Index, although the degree of obstructive sleep apnea and tiredness during the day are greatly reduced after MBS, most patients still had the condition at monitoring based on the standard criteria [148].

It has been often documented that MBS improves OSA. According to Buchwald and colleagues’ analysis, 85.7% of patients had their OSA resolved, which is a significant improvement for the patient group as a whole [149]. These findings imply that patients after MBS should have ongoing monitoring for potentially persistent, clinically significant obstructive sleep apnea and should receive appropriate treatment, taking into consideration symptoms and coexisting medical conditions.

### 7.5. The Management of Osteoarthritis

Obesity is one of the primary causes for osteoarthritis in the hip and knee [150,151]. Although MBS is a successful obesity treatment that leads to weight reduction over time, it is unclear if this procedure lowers the likelihood of osteoarthritis in the knee and hip and lessens the need for arthroplasty. In the first year following MBS, many patients with severe obesity may see improvements in their functional status and knee pain, according to observational studies. However, more thorough, long-term research is required to corroborate these results [152].

Uncontrolled research suggests that MBS may reduce cartilage turnover markers, reduce the symptoms of osteoarthritis in the knee, and potentially delay the need for arthroplasty. Patients receiving MBS who had radiographic evidence of knee osteoarthritis and symptoms were included in a 5-year prospective observational study. The average rate of total weight reduction maintained by the patients was 22.3% at the 5-year follow-up, according to the results (*p* < 0.001). Pain, stiffness, and physical function, assessed at six months, a year, and five years, showed statistically significant improvements from baseline. The conclusions were that at a five-year follow-up, MBS is an effective and safe way to help people with knee osteoarthritis maintain their weight loss, have less pain and stiffness, and have better function [153].

Nine observational studies assessing the immediate or long-term results of total joint replacement in 32,985 people who had never had MBS and 5743 post bariatric patients were investigated in a recent systematic review. Reductions in the length of stay, surgical time, and short-term medical problems were linked to MBS. However, MBS did not correlate with decreased risk over time for venous thromboembolism or wound infection from arthroplasty, nor did it correlate with decreased potential risks for dislocation, periprosthetic infection, periprosthetic fracture, or revision [154].

### 7.6. The Impact on Fertility

Obese women are predicted to have a 5% lower likelihood of becoming pregnant for each BMI unit above 29 kg/m^2^, with a three-times-bigger relative danger of infertility than normo-ponderal women [155]. Weight loss with MBS may improve ovulatory dysfunction and irregular menstruation while promoting spontaneous conception. Within 2.5 years of follow-up, 69% of infertile obese women were able to conceive [156,157].

Following MBS, improvements in women’s periods and endocrine balance are immediately noticeable, and infertile women may become spontaneously pregnant as a result of these changes [158]. Additionally, there is an abrupt rise in the level of the sex hormone binding globulin (SHBG), a drop for testosterone level and an increase in follicular stimulating hormone (FSH). Another improvement in the menstrual cycle is a decrease in follicular phase length after gastric bypass surgery. These outcomes were shown in prospective research which included 29 obese pregnant women and was conducted over a period of three years [107].

A study published in 2021 assessed the impact of the type of MBS on pregnancy outcomes. The outcomes showed that, compared to gastric bypass, women who became pregnant following sleeve gastrectomy and gastric banding had a decreased risk of anemia (*p* < 0.05). Despite women who underwent gastric banding having much higher weights at booking and experiencing prenatal weight gain, their chance of developing diabetes during pregnancy was lower after gastric banding than following gastric bypass (*p* = 0.03). It was also shown that women who underwent gastric banding gave birth to babies who were heavier than women who underwent gastric bypass (*p* < 0.001). Women who had gastric banding had a greater risk of premature birth than those who had gastric bypass surgery (*p* < 0.05) [159].

### 7.7. Urinary Incontinence

Urine incontinence is known to be associated with obesity. The mechanical and metabolic factors are involved in the etiology of this condition. In an observational study at ten medical units, patients undergoing MBS were recruited. A total of 1987 (80.8%) of the 2458 participants finished the baseline and follow-up questionnaires. The frequency of urinary incontinence was greater among women than men (*p* < 0.001). The prevalence of the urinary incontinence, after a year, was considerably lower in males (9.8%) and women (18.3%) while the weight loss was 27.0% in men and 29.5% in women (*p* < 0.001 for all). The 3-year prevalence was significantly lower than at the beginning (*p* < 0.001 for each parameter), but it was greater than the 1-year prevalence for both men and women (25% among women and 12% among men) [160].

## 8. Risks Associated with MBS

### 8.1. Early Complications of MBS

#### 8.1.1. Intestinal Obstruction and Stenosis

Stoma blockage following an adjustable gastric band operation is not uncommon. The mechanical obstruction could result from the extra gastric band eroding into the gastric wall or from the band slipping and the distal section of the stomach herniating through it. Operative correction is necessary for several kinds of stoma obstructions connected to bands [161].

After laparoscopic rather than open bariatric operations, internal hernias are more likely to happen. A large portion of the small intestine may be in danger due to intestinal obstruction and strangling caused by internal hernias. The most precise examination technique appears to be computed tomography, but radiographically finding these hernias may not always be easy [162].

After RYGB, stenosis can occur in 8% to 19% of cases, with a higher frequency occurring following anastomoses completed with an end-to-end anastomosis stapler [163].

In terms of frequency, diagnosis, and treatment, stenosis following an SG is not the same as RYGB stenosis. True stenosis or stricture after an SG is rare, occurring in 0.69% to 2% of individuals [164,165].

#### 8.1.2. Gastrointestinal Bleeding and Perforation

Intestinal hemorrhage is a potential perioperative challenge after RYGB surgery. Mild to moderate hemorrhage from peripheral ulcers occurs in 5% of individuals; serious bleeding is much less common. Approximate 11% of people who underwent RYGB and SG experience postoperative bleeding that needs to be addressed [166].

One known adverse consequence of the surgery interventions for obesity is marginal ulcers. A systematic review from 2022, on 610 patients, from 26 articles, showed that there was a 27.5 ± 8.56-month interval between the initial MBS and the perforated marginal ulcer (PMU) diagnosis. Abdominal discomfort was the most prevalent presenting symptom (99.5%), and 72% of the patients underwent a computed tomography scan as their diagnostic method. At the time of perforation, only 15% of patients were using prophylactic H2 blockers or proton pump inhibitors, and 41% of the participants smoked. Of the patients, twenty-three percent were taking nonsteroidal anti-inflammatory medications. The conclusion was that following a gastric bypass, PMU is a surgical emergency that carries a high risk of serious illness or even death [167].

For assessing the frequency and management of bleeding issues following gastric bypass surgery, a study from 2012 included 4466 patients who experienced gastric bypass surgery over a 10-year interval for which accurate morbidity data were available. A total of 42 (94%) of all patients had a bleeding problem after surgery. Twenty (47.6%) of these patients had had prior abdominal surgery. Thirty patients (71%) experienced bleeding on the first postoperative day (<30 d). The hemorrhage was caused by either mesenteric vascular bleeding, iatrogenic visceral damage, or bleeding from the staple lines. In 43% of cases, early postoperative bleeding necessitated surgical intervention to achieve hemostasis. Thirty-three percent of cases of late postoperative hemorrhage (n = 12) required surgical intervention due to marginal ulceration. In 14.3% of cases, previously undetected bleeding diatheses were found [168].

#### 8.1.3. Postoperative Gastrointestinal Leaks

Postoperative gastrointestinal leaks are still a relatively rare complication with a significant chance of serious complications and death. A chart review study showed that the incidence of leaks following gastrointestinal procedures varies according to the anastomosis site. For example, the esophagus leak frequency is found to vary from 2 to 16%, the stomach from 1 to 9 percent, the pancreas from 9 to 16%, the bile ducts from 10 to 16%, the small intestine from 1 to 3 percent, the colon from 3 to 29%, and the rectum from 8 to 41%. The study monitored patients post MBS to evaluate their survival for a maximum of ten years after the surgery. Those with gastrointestinal anastomotic leaks were compared to those whose anastomoses remained intact in terms of morbidity, mortality, and cost. Gastrointestinal leaks can cause up to 35% of deaths [169].

Chang et al. assessed thirty-day major complications following bariatric procedures. This analysis included 107,874 patients from 71 American studies conducted between 2003 and 2014. The patients experienced SG, adjustable gastric banding, or gastric bypass. Among surgical techniques, the anastomosis’s rate of leaks lasting fewer than 30 days was 1.15%; the rate for SG was higher than that of gastric bypass surgery (1.14%) at 1.21% [170].

#### 8.1.4. Thromboembolism

A 2013 US study sought to determine how often deep vein thrombosis (DVT) and/or pulmonary embolism (PE) occurred in participants having MBS. Out of 508,230, 4500 people had PE (0.9%). A total of 6480 of 508,230 cases (1.3%) of DVT without PE and 10,980 cases (2.2%) of VTE (either PE or DVT) were reported. The number of PE patients who died in hospital was 130 out of 508,231 (0.03%) [171].

Gonzales et al. wanted to identify those patients at the greatest risk for DVT/PE for preventing these events after MBS. They collected data from 660 patients who previously underwent RYGB. Statistically significant results were obtained showing that anastomotic leak, smoking, age over 50, and a history of DVT or PE all raise the risk of postoperative thromboembolic complications [172].

Other studies demonstrated that many thromboembolic events happen three weeks after the procedure, but there is no data or agreement on the ideal amount of time to provide chemoprophylaxis. Patients having open procedures or revision bariatric surgeries, persons with a BMI above 50 kg/m^2^, patients undergoing longer than four hours of surgery, patients with hypercoagulable states, and patients with obesity hypoventilation syndrome are the patients most susceptible to develop VTE, although opinions on the risk to these patients are divided [173,174].

### 8.2. Late Complications of MBS

#### 8.2.1. Cholelithiasis

After MBS, cholelithiasis is a severe consequence that requires careful observation. The hepatic circulation of bile disruption causing excessive absorption of cholesterol or super saturation of cholesterol; intestinal dysfunction leading to rapid growth of cholesterol particles and solid fat particles; and family history causing excessive hepatic cholesterol secretion are the four main causes of cholelithiasis [175].

Ironically, gallstone formation is a common postoperative concern for bariatric patients, ranging from 30% to 53%. The rising rate of cholelithiasis following MBS could be attributed to two factors. One is that fast weight loss raises blood cholesterol and triglyceride levels by causing fat to be mobilized. On the contrary, decreased cholecystokinin levels brought on by intestinal dysfunction following MBS may result in gallbladder contractile dysfunction [176,177].

Sweden was conducting a population-based cohort study on MBS, between 1980 and 2010, on 8910 patients who had not previously received treatment for gallstone disease and had undergone obesity surgery. The study showed that the surgical group experienced a higher incidence of cholecystectomy (106 vs. 19.5/10,000 person-years) in contrast to the control group. Gallstones reached the incidence ratio of 5.5 (range 5.0–5.9), while cholecystectomy had an incidence ratio of 5.4 (range 5.0–5.9). Therefore, cholelithiasis incidence in MBS patients was five times greater than in the overall population [178].

King Saud University Medical City performed a retrospective study from 2016 to 2018 on 711 patients who experienced LSG and were between the ages of 18 and 60. The results revealed that 3.5% of patients had symptomatic cholelithiasis after surgery. The average time for the onset of symptoms was 12.4 months. Patients with symptomatic cholelithiasis experienced a significantly higher prevalence of weight loss at six and twelve months (28.94 ± 4.89% and 38.51 ± 6.84%, respectively; *p* = 0.002) compared to patients without symptomatic cholelithiasis (24.41 ± 6.6% and 32.29 ± 10.28%), respectively; *p* = 0.012 [179].

Much research is based on RCTs examining the prophylactic efficacy of ursodeoxycholic acid (UCDA) on cholelithiasis following MBS. A randomized control trial from Egypt involved 1432 morbidly individuals with obesity previously treated with greater curve plication (GCP), SG, or laparoscopic OAGB. They were divided into two groups at random and given either UDCA or a placebo, with a one-year minimum further investigation period for the evaluation of cholelithiasis and weight. It was assessed that after surgery, the incidence of cholelithiasis was 9.7%. The incidence of gallstone development significantly decreased in the UDCA-treated group, going from 22% in the placebo group to 6.5%. Compared to non-developers, gallstone patients had a significantly higher mean proportion of excess lost weight (%EWL). A statistically significant 64.7% of individuals developing gallstones had SG, compared to 28% in OAGB and 7% GCP. Six is the NNT, 70.4% is the AR%, and 3.4% is the RR in preventing cholelithiasis [180].

#### 8.2.2. Nephrolithiasis

Nephrolithiasis with a new onset has historically been associated with MBS; the average interval between resection and nephrolithiasis diagnosis is 1.5 to 3.6 years [181]. Its incidence varies with type of procedure: it is greatest (22–28.7%) for malabsorptive procedures, medium (7.65–13%) for RYGB procedures, and lowest (LAGB, LSG) for simply restrictive techniques, where it is comparable to that of overweight controls undergoing non-surgical procedures [182,183].

A study carried out on 201 patients which assessed the possibility of upper urinary tract calculus diagnosis or treatment following gastric banding demonstrated that the percentage of kidney stone recurrence in RYGB patients with experience of gallstones was 18.6% (27/145 patients) within approximately two years after RYGB, in contrast to 8.6% (534/6390) of patients without previous experience of gallstones. The findings are limited because only four small groups have shown incidences of recurrent stone sickness after RYGB in comparison to approximately 6400 individuals who had never had a stone. During a comparable 2-year period, the incidence of stones was 1.3% (8/618) for restrictive operations (LAGB and SG) and 4.6% (258/5569) for obese controls [184].

In these patients, urinary metabolic abnormalities including decreased urine volume, hypocitraturia, and hyperoxaluria lead to nephrolithiasis. Generally speaking, rather than secondary hyperoxaluria, which is linked to increased intestinal absorption or excessive oxalate intake, primary hyperoxaluria arises from uncommon monogenic illnesses. Excessive hepatic synthesis of endogenous calcium due to different mutations is the hallmark of primary hyperoxaluria. Secondary hyperoxaluria is the most frequent metabolic abnormality among persons who have had MBS, with rates of incidence between 29% and approximately 67%, at three months and two years after MBS [185].

Even though a low-oxalate diet is advised to avoid hyperoxaluria and gallstone development after bariatric interventions, it may be challenging to restrict oxalate in the diet because people may not be aware of how much oxalate is in different foods. The foods that contain the highest amount of oxalate are beans, soy, dark tea, parsley, some berries, spinach, rhubarb, beets, starfruit, and nuts [186].

Although consuming meals rich in fruits and vegetables might help meet this need, ascorbic acid pills have been extensively taken by the general population. But epidemiological statistics indicate that consumption of vitamin C tablets (>1000 mg/day) were correlated with a 16% rise in the prevalence of kidney stones in men and an increase in oxaluria [187]. Research demonstrated that, in the case that prospective examinations of a greater number of post-BC patients corroborate the occurrence and severity of intestinal hyperoxaluria, all patients may require prophylactic therapy techniques after the procedure. Meanwhile, all patients who get renal stones following MBS should have a metabolic evaluation and start a stone-prevention medication [188].

#### 8.2.3. Bone Loss and Fracture Risk

MBS patients have a comparatively greater long-term risk of bone loss due to their fast weight loss, reduced nutritional intake, and impaired absorption of micronutrients. After MBS, there is an increased risk of fracture and bone demineralization. Deterioration of bone health following surgery can be attributed to decreased mechanical loading, altered adipocyte and gastrointestinal hormone levels, and malabsorption, namely of calcium and vitamin D [189].

Impaired intestinal calcium absorption is a major contributor to bone loss because it triggers the production of parathyroid hormone (secondary hyperparathyroidism) and causes bone resorption [190].

Patients with MBS are relatively more prone to bone loss over the long term because of rapid weight loss, reduced food consumption, and impaired absorption of nutrients through many pathways [191].

Published research indicates that people who undergo obesity operations (such as RYGB and BPD) that alter food absorption are more vulnerable to deteriorating bone health than those who undergo restrictive surgeries (like SG and AGB). Exercise and dietary modifications that increase calcium, vitamin D, and protein intake are the most important methods for treating this problem, according to studies. Additionally, looking at some of the elements we will go over below can benefit doctors and patients both before and after surgery. If there is a vitamin or micronutrient deficit, they can take the appropriate measures [192].

Though calcium can be absorbed throughout the stomach, it is not just absorbed in these areas, and people having RYGB may have sufficient calcium absorption. According to a study that looked at how RYGB affected intestinal fractional calcium absorption (FCA), even with calcium intake and a level of 25(O. H.) D ≥ 30 ng/mL, FCA drastically drops following RYGB. For preventing diseases caused by calcium and to preserve calcium homeostasis, the authors recommended that the patients increase their consumption of calcium. Approaches to calcium supplementation after MBS generally require more research [193].

#### 8.2.4. Steatorrhea

Testing for pancreatic exocrine deficiency, coeliac sprue, and bacterial overgrowth is necessary in cases of steatorrhea following bariatric procedures. An abnormally short common channel, meaning of the distal part of the small intestine where meals and biliopancreatic fluids combine, is most likely the cause of steatorrhea. This scenario can be created by surgical blunders [194].

A non-clinical trial explored how dietary fat and oxalate affected urine parameters and fecal fat excretion in a rat model of RYGB surgery. The outcomes showed that high fat feeding in this RYGB model was the cause for steatorrhea, hyperoxaluria, and low urine ph. The amount of oxalate excreted by RYGB rats on normal fat and oxalate-free nutrition was twice that of age-matched, sham-operated controls. While nutrition and the gut seem to be the primary mediators of RYGB hyperoxaluria, additional research is necessary to determine the role of the liver or other processes as secondary sources of oxalo-genesis [195].

**Figure 4:**
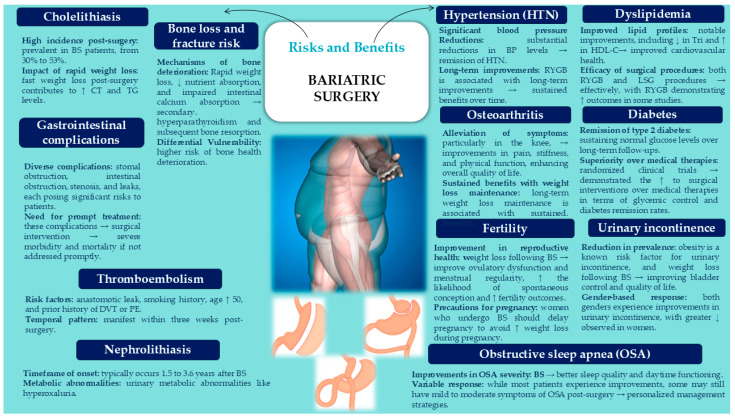
Risks and benefits of MBS.

Figure 4 briefly presents the risks and benefits related to MBS.

## 9. Weight Regains After MBS

Despite the many benefits of MBS, research shows that a significant proportion of patients experience weight regain over time. After patients attain their nadir weight, weight regain (WR) is common; approximately 20–25% of patients experience significant WR following MBS [196,197].

Data on pre and postoperative weights during a 5-year period were gathered in multicenter research, including many patients. The percentage of patients with a considerable weight gain (more than ten kg) from nadir, a weight gain of 25% of lost weight from nadir, and a BMI regain of more than 5 kg/m^2^ from nadir was determined using several criteria. Across subgroups, the percentage of individuals with significant WR was compared. The result was that from the 9617 patients included in the study, 5 kg, 1.92 kg/m^2^, and 14.1% of lost weight were the median weight loss after five years [198].

A total of 37% of three hundred RYGB subjects, from another research study, experienced significant weight gain at the 7-year follow-up, which was defined as a rise of at least 25% from the nadir weight [199].

Ten years after surgery, patients had regained between 20 and 25% of their lost weight, as SOS research showed. Patients who underwent RYGB had a weight gain of 12% of their total body weight, but those who had S experienced varying weight gains, varying from 6% in the two years following surgery to 76% six years afterwards [200].

Variables following surgery linked to weight gain were a bigger gastrojejunal stoma diameter, greater gastric volume after SG, more monitoring time after the intervention, diabetes, bulimia, increased meal impulses, excessive nighttime eating, less physical activity, lower social support, stressful living, consumption of alcohol, and symptoms of depression [201]. Furthermore, although the evidence is limited, weight regain was linked to increased pre-prandial ghrelin and decreased postprandial GLP-1 levels [202]. But for now, there is no established standard treatment for gaining weight after MBS. Typically, medication is used in conjunction with extra behavioral interventions to promote lifestyle changes. Glucagon-like peptide-1 receptor agonists, or GLP1-RAs, are the active substances that reduce weight most effectively right now. Liraglutide and semaglutide are two GLP1-RAs with a well-documented safety profile and efficacy that includes average weight loss of up to 15%, mild to moderate gastrointestinal adverse reactions that are often temporary, and advantages to the kidneys. It is unclear how they should be used to manage weight gain following MBS [203,204].

In comparison to other combination anti-obesity medications, phentermine/topiramate was linked to the highest chance of achieving 5, 10, and 15% weight loss, according to a comparable study with 197 post-surgical patients [205]. When phentermine 37.5 mg and phentermine/topiramate 7.5–46 mg were compared in a retrospective study including 30 patients, both resulted in statistically significant weight decreases over a 90-day period of 6.3 kg and 3.8 kg, respectively. However, phentermine 37.5 mg induced greater weight loss [206].

## 10. Psychological Implications and Body Image After MBS

### 10.1. Psychological Alterations After MBS

Individuals with obesity may experience psychological distress as a result of social stigma and self-potential humiliation, which may worsen with the increase in BMI [207].

Particularly in MBS candidates, there is mounting evidence of a reciprocal relationship between obesity and mental illness. Obesity frequently coexists with common mood disorders such anxiety and depression. The incidence of depression in obese patients undergoing surgery is higher than the reported prevalence in the general US population (19% versus 8%) [208].

Following MBS, the majority of people have a significant increase in their psychological functioning. Researchers classified 69 patients undergoing bariatric surgery as having “great psychosocial stress” or “little” or “no psychosocial stress” using four scales with clinical threshold scores for depression, anxiety, relationships, and binge eating. The incidence of severe psychosocial stress dropped from 70% to 12% ten months following surgery [209].

It has long been believed that binge-eating disorders and depression are closely related. Patients with eating disorders are more burdened than those who are obese [210]. MBS has been demonstrated to reduce physical health issues, but its effects on mental illness have not been well-defined. Nevertheless, long-term follow-up data revealed that some surgical patients did not perceive psychological improvements or higher rates of depression [211].

Many are concerned about the potential risks of mental health conditions such substance abuse, suicide, and self-harm, after MBS. One study found that patients with BS were 1.98 times more likely to commit suicide than those who received normal treatment for their adiposity [212]. On average, 3.8 to 3.9 years following surgery, suicide and suicide attempt rates occur [213].

A very recent review included the results from nine studies which had previously studied the mental disorders associated with bariatric surgery. The results showed that main mental health issues associated with BS were depression syndrome (in 50% of the cases), alcohol use disorders in 15% of the cases, suicide, self-harm, binge eating disorders, eating disorders, anxiety symptoms, and general mental health—each with an incidence of 5% among patients who had gone through a bariatric procedure [214].

### 10.2. Body Image After BS

When talking about body image, the term implies different aspects (i.e., cognitive, affective, behavioral, and perceptual). About one in five individuals undergoing MBS has stated that their main reason for having surgery is appearance. Although the impact of several bariatric procedures on weight reduction and metabolism have been extensively studied, little is known about the complications of body image after surgical intervention [215].

Following successful MBS, weight reduction frequently involves extra skin on all parts of patients’ bodies, including the upper arms, thighs, flanks, buttocks, chest, and abdomen. In addition to being unsightly, this extra skin can cause severe and recurring intertriginous rashes, stasis dermatitis, ulcerations, and ulcers that frequently do not improve with medication [216].

The majority of doctors and surgeons concur that both reconstructive and cosmetic procedures should be carried out no sooner than 18 to 24 months following MBS and at least 6 months following weight stability. In order to maximize results, maintain patient safety, and accommodate reconstruction operations specific to them following MBS, patients may need more than one body reshaping intervention [217].

As a result, after BS, some patients have chosen to have body-contouring surgery (BCS). Up to 84.5% of patients who had bariatric surgery want to have another BCS; the rates are higher for women than for males, with 75% and 68%, respectively. The progression of body image issues before and after bariatric surgery, as well as in relation to BCS, are little understood [218,219].

Those with previous experience of bariatric surgery seeking BCS reported higher levels of negative body image across the range of body image measures than the general population and a non-surgical control group matched on BMI and demographic variables. When compared to post-bariatric individuals without BCS, those who received BCS reported improvements in several body image indices, including physical functioning, body area satisfaction, and appearance appraisal, even after adjusting for weight reduction and time since surgery. After receiving BCS, some patients still voice dissatisfaction, nonetheless [220].

Furthermore, 27.2% (n = 15) of respondents said they were “very dissatisfied to dissatisfied” following an abdominoplasty, while 40% (n = 6) said they were “very dissatisfied or dissatisfied” following a thigh lift. It is interesting to note that none of the 10 people who had breast lifts expressed unhappiness. Furthermore, patients expressed ongoing discontent with non-contoured parts [221].

## 11. Conclusions and Future Perspectives

For people with a BMI higher than 40 kg/m^2^ or 35 kg/m^2^, with obesity-associated diseases, MBS is a highly effective method of therapy. When nonsurgical measures are ineffective in controlling the patient’s weight, surgical intervention becomes essential. When compared to non-surgical therapy, research shows that MBS lowers mortality and morbidity rates in individuals with obesity. The majority of those with OSA, diabetes, hyperlipidemia, and high blood pressure saw a complete resolution of their symptoms or, at least, a significant improvement. However, the effectiveness of MBS depends not only on the type of surgical method used. An interdisciplinary approach is required to meet the patient’s new demands. One of the main components of this multidisciplinary approach is the collaborative work of a team consisting of different medical specialists (i.e., surgeons, clinical pharmacists, nurses, clinical psychologists, dietitians, cardiologists, and diabetologists).

Additional research is necessary since long-term patient sustainability of the results obtained after the surgical interventions represents an essential component. Gaining an in-depth understanding of the sustainability over time of weight loss, metabolic advantages, or possible disadvantages is essential to support therapeutic decision making. Data about the frequency of persistent symptoms and recurrences of obesity are still lacking. Although losing weight and improving metabolism are still important, understanding the wider effects of these surgical procedures, on things like general quality of life, psychological health, and patient satisfaction, is just as important.

## Figures and Tables

**Table 1:** Metabolic bariatric surgery updated criteria and recommendations.

Criteria for MBS		Recommendations
BMI	30–34.9 kg/m^2^	Individuals suffering from type 2 diabetes.
		One medical issue linked to obesity.
		Patients whose nonsurgical weight reduction or co-morbidity improvement is not significant or long-lasting.
	35–40 kg/m^2^ without comorbidities	Individuals with a BMI of over 35 kg/m^2^, independent of the existence, degree, or absence of problems associated with obesity.
	For Asian population	Patients with a BMI over 25 kg/m^2^ are considered to be clinically obese in the Asian community; the conventional BMI requirements alone should not be used to deny someone access to MBS.
Extreme age	Elderly	MBS is linked to somewhat greater rates of postoperative problems in septuagenarians than in younger people, but it still offers significant advantages in terms of weight loss and the remission of comorbid diseases.
		Indications for MBS include fragility, mental status, smoking status, and end-organ functionality.
		An age restriction for older individuals seeking MBS is not supported by any data, although it is advised to carefully choose patients.
	Pediatrics and adolescence	Both normal growth and pubertal development are unaffected by MBS.
		MBS provides long-lasting weight loss and reduces comorbidities, and it is safe for people under the age of 18.
Link to other therapies	MBS before a joint replacement	Orthopedic surgical societies recommend avoiding hip and knee replacement for patients with a BMI more than 40 kg/m^2^ due to the greater likelihood of recurrence and surgical complications, such as wound infection and deep vein thrombosis.
		Before total knee and hip replacement, MBS has decreased the length of hospital stays, surgery times, and early postoperative complications.
		For an individual with a BMI of over 30 kg/m^2^, MBS is recommended before joint arthroplasty.
	Treating abdominal wall hernias and MBS	One risk factor for the occurrence of ventral hernias is obesity.
		To lower the risk of postoperative complications, MBS is advised before to ventral hernia repair, in patients with obesity and an abdominal wall hernia.
	MBS before receiving an organ transplant	Obesity may restrict opportunity to transplantation and is linked to end-stage organ disease; obesity presents special technical difficulties during surgery and is a relative contraindication for solid organ transplantation.
		According to published data, people with grade 3 obesity and end-stage renal illness may be eligible for a kidney transplant following MBS.
		In certain individuals who would not otherwise be eligible, MBS has been demonstrated to be a safe and successful gateway to liver transplantation.
		MBS may increase lung transplant eligibility.
		MBS can be performed simultaneously or after solid organ transplantation, to lower mortality and complication rates.
		MBS can enhance the results of heart transplants.
Patients with high risk factors	BMI ≥ 60 kg/m^2^	MBS is both safe and effective.
		Research indicates that individuals with a BMI bigger than 60 kg/m^2^ are more likely to experience perioperative problems following MBS.
		The evidence shows that MBS is safe for patients whose starting BMI was 70 kg/m^2^.
	Individuals with liver cirrhosis	A major risk factor for both liver cirrhosis and metabolic dysfunction-associated liver disease is obesity.
		MBS has been linked to liver fibrosis regression and histologic improvement in metabolic dysfunction-associated liver disease.
		MBS is linked to a lower risk of MAFLD developing into liver cirrhosis.
		High perioperative mortality is linked to MBS in patients with “decompensated” cirrhosis. To guarantee the greatest results, careful patient selection and surgical method selection are crucial.
	Individuals with heart failure	A major risk factor for both liver cirrhosis and metabolic dysfunction. MBS is linked to improved left ventricular ejection fraction, increased functional capacity, and an increased risk of heart transplantation in individuals with obesity with heart failure.
		MBS has a low morbidity and death rate in individuals with HF and obesity, and it may be a helpful adjuvant prior to left ventricular assist device implantation or heart transplantation.
Assessment of the patient	Interdisciplinary treatment	In preoperative and postoperative care of MBS patients, interdisciplinary treatment plays a crucial role.
	MBS adjustment	Adjustment surgery following MBS may be indicated for a variety of reasons: inadequate weight reduction or regain, inadequate co-morbidity remission, and the treatment of comorbidities, such as acid reflux.
		Adjustment surgery following MBS might be linked to increased perioperative difficulties. Still, it can result in acceptable mortality and morbidity rates together with satisfactory metabolic outcomes.

MBS, metabolic and bariatric surgery; BMI, body mass index; MAFLD, metabolic dysfunction-associated fatty liver disease; HF, heart failure.

**Table 2:** Extreme categories of population—expectations and risks.

Categories/Age	Type of MBS	Results	Risks	Ref.
Older population ≥ 70 years old	LSG	Following an average follow-up of 31.3 and 33.5 months, 24.6 percent of the total body weight was lost. Remission of co-morbidities.	MBS is linked to a slightly increased incidence of complications following surgery.	[59,60]
Pediatrics and adolescents/ ≤18 years old	RYGB	Significantly better. Improvement in cardiovascular co-morbidities and weight loss as compared to adolescents receiving medication care. Improvements in dyslipidemia and hypertension have been shown for up to eight years following surgery. Substantial loss of body weight and sustained reductions in cardiovascular risk factors and type 2 diabetes.	Dietary deficits, incisional hernias, failure of therapy resulting in surgical correction, and reflux gastroesophageal reflux are chronic issues that are most described.	[61,62,63,64]

LSG, laparoscopic sleeve; MBS, metabolic and bariatric surgery; RYGB, Roux-en-Y gastric bypass.

**Table 3:** Physiologic changes to the digestive system following MBS.

Affected Gastrointestinal Physiology/ What Changes	Mechanism of Action	Physiologic Role	Impact of MBS	Ref
Cephalic phase/ Sight, smell, thought or taste of food	Influencing the levels of ghrelin, insulin, PP, and gastrin	Ghrelin, an orexigenic hormone released by the stomach before a meal, increases appetite.	Initially suppressed ghrelin amount, but months following surgery, restore to their preoperative values.	[66,67,68]
		Pancreatic polypeptide, a pancreatic islet-expressed anorexigenic hormone that is released in response to dietary stimulus and necessitates intact parasympathetic vagal nerve system signaling.	Following RYGB and LSG, PP has typically been reported as stable; however, some studies have found a decrease in fasting levels following RYGB.	[69,70,71]
		Gastrin, mostly released by the stomach’s antrum’s G-cells, it helps release gastric acid and may also aid in the production of insulin through the islets of the pancreas’ gastrin receptors.	Gastrin levels are unchanged in some studies. According to one study, two weeks after RYGB, postprandial levels were reduced. According to a study on mice, postoperative weight reduction may be attributed to a decrease in gastrin following RYGB.	[72,73,74,75,76]
Chewing and tasting/Chewing time, taste preference and food perception	Elevated concentrations of the satiety hormones GLP-1 and PYY.	The release of gastrointestinal hormones that are essential for energy homeostasis, food intake, and satisfaction is regulated by signals that the brain receives from the sense of taste and gastrointestinal membrane.	A modification in flavor and a lack of hunger. A selective decrease in brain reactions to high-calorie foods could be the cause of this. Longer chewing durations and more chewing cycles while eating solid meals. A reduction in meal size.	[77,78,79,80,81]
Gastric phase/Emptying the stomach entero-gastrointestinal transit time and values	Increasing or decreasing the stomach emptying time.	Emptying the stomach and intestinal transit time are controlled by hormonal, neurological, and stomach contents.	Slower stomach emptying time for solids but faster gastric emptying for liquids. Higher levels of glucagon and quicker transit time. Accelerated gastric emptying and shortened transit time.	[82,83,84]
Phase of the intestine and gut peptide/ The expression of OXM, PYY, and glp-1, the gut hormones that cause anorexia	Enhanced	GLP-1 released from the colon’s and small intestine’s distal l-cells, insulin production rises while glucagon, other gastrointestinal secretions, and motility are reduced. GLP-2 secreted from intestinal l-cells following meal consumption; it increases the absorptive surface area of the ileal and colon mucosa by promoting cellular proliferation and inhibiting apoptosis. GIP produced in the jejunal and duodenal mucosa by k-cells.	The levels during meal stimulation or oral glucose supplementation have been demonstrated to be constantly boosted following MBS. It has been unpredictable following MBS; certain investigations have shown a rise in these levels, while others have shown a fall.	[85,86,87,88,89,90]
		PYY is released by the small and large intestine’s mucosal L-cells and suppresses intestinal, pancreas, and stomach secretions.	The results of the studies are controversial; some reported increased postprandial PYY3-36 after MBS.	[75,88]
		OXM is an anorexigenic peptide that intestinal L-cells co-secrete along with PYY and GLP-1.	Postprandial OXM rises 1–2 months following RYGB.	[91]
		CCK-GE/stomach motility is inhibited by this satiety hormone, which is generated by intestinal I-cells.	The levels of CCK increase after MBS.	[92,93]
Absorbative phase/Nutrient absorption	Diminish	This occurs subsequently to the metabolism of vitamins, minerals, proteins, lipids, and carbs—all of which are necessary for cellular repair, development, and the synthesis of energy.	Micronutrient deficiencies can include iron, selenium, zinc, and copper as well as vitamins A, C, D, K, thiamine, folic acid, and B12; these deficiencies are frequently linked to RYGB and BPD.	[94]
Ileal break/Gut hormones on ingestive behavior mediated by neuroendocrine mechanism	Activating the ileal break which causes a reduction in jejunal contraction, delayed GE, and elevated ITT, all contributing to extended satiety; PYY, GLP-1, and possibly OXM could act as mediators in the ileal brake.	The ileal brake is a distal-to-proximal unfavorable reaction mechanism that effects jejunal motility, ITT, GE, and pancreatic and biliary contents.	It is unknown how much the ileal brake contributed in relation to the metabolic benefits seen in the context of MBS.	[95]
Liver and bile acid phase/The transport of nutrients from the bloodstream to the liver and glucose metabolism	Hypothalamic metabolic centers are activated by glucose in the portal vein; consequently, consumption of food is reduced and the insulin sensitivity and equilibrium of glucose enhanced (due to suppressed HGP).	The removal of cholesterol comes first and is the most crucial step; by transforming cholesterol into bile acid and causing the cholesterol in bile to become micellar soluble, bile acids help the body remove cholesterol by allowing it to pass from the hepatocyte into the intestinal lumen and eventually be eliminated through the fecal pathway.	When weight loss occurred, RYGB and LSG dramatically enhanced insulin sensitivity and glucose metabolism in rat research employing a hyperinsulinemia glycemic clamp. Following RYGB, nondiabetic obese adults showed better hepatic insulin index, high levels of insulin and C-peptide, and similar natural synthesis of glucose in comparison with the slim and obese control subjects. Subjects with type 2 diabetes showed improved hepatic metabolism one month after RYGB, as seen by improvements in their hepatic insulin sensitivity index and HGP, without corresponding improvements in their peripheral insulin sensitivity.	[96,97,98,99]
Large intestine and microbiota phase/Food digestion and the balance between the various bacterial families of the microbiota	The colon adjusts and can function as a digestive organ, breaking down partially absorbed proteins and carbs through bacterial fermentation; this is then absorbed and contributes in some way to the body’s energy supply.	Water and electrolytes are primarily absorbed in the large intestine. Several metabolic activities, including the generation of vitamins and amino acids, the degradation of indigestible carbs, and the biotransformation of BA, are carried out by the vast population and variety of bacteria found in the large intestine.	Both obese/normal-weight patients showed increased Firmicutes microflora, which decreased after RYGB; post-surgery, there was a notable rise in Gamma proteobacteria, absent in pre-surgery. Patients with RYGB who took probiotic supplements saw a higher percentage of excess weight reduction six/twelve weeks following surgery.	[100,101,102,103]

RYGB, Roux-en-Y gastric bypass; LSG, laparoscopic sleeve gastrectomy; PP, pancreatic polypeptide; GLP-1, glucagon-like peptide-1; PYY, peptide YY; GE, gastric emptying; ITT, intestinal transit time; OXM, oxyntomodulin; GLP-2, glucagon-like peptide-2; GIP, gastric inhibitory peptide; CCK, cholecystokinin; MBS, metabolic and bariatric surgery; BPD, biliopancreatic diversion; HGP, hepatic glucose production.
